# Lateral-dock single-port robotic-assisted extended totally extraperitoneal plasty (eTEP)-Sublay-Herniotomy-Procedure– presentation of a novel technique for robotic-assisted ventral hernia surgery (with video)

**DOI:** 10.1007/s10029-025-03420-w

**Published:** 2025-07-12

**Authors:** Julian Hipp, Robin Klewitz, Hannes Neeff, Stefan Fichtner-Feigl, Philipp Anton Holzner

**Affiliations:** Department of General and Visceral Surgery, Medical Center, University of Freiburg, Hugstetter Str. 55, 79106 Freiburg, Germany

**Keywords:** eTEP, Robotic surgery, Ventral hernia repair, DaVinci SP-system, Single port-robotic surgery

## Abstract

**Introduction:**

Robotic-assisted minimally-invasive extended totally extraperitoneal plasty (eTEP)-sublay-herniotomy is one of the most promising novel techniques for the management of ventral hernia. While several techniques for multiport-robotic-assisted eTEP have been described, only very few reports on suprapubic single-port robotic-assisted eTEP-techniques have been published. The technical limitations of this access leave room for further technical development using single-port-robotic systems.

**Methods:**

We give a detailed description of our novel lateral-dock single-port robotic-assisted eTEP-procedure (Freiburg approach, FReTEP). Feasibility of the access was demonstrated within a human cadaveric procedure, and two consecutive patients were treated using the FReTEP-procedure.

**Results:**

Two consecutive patients were successfully treated without postoperative complications and without early hernia recurrence using the FReTEP-procedure.

**Conclusion:**

The FReTEP-procedure is a promising novel technique for single-port-robotic-assisted ventral hernia repair. Further studies are needed to evaluate the novel procedure.

**Supplementary Information:**

The online version contains supplementary material available at 10.1007/s10029-025-03420-w.

## Introduction

Primary ventral hernia and incisional hernia are a common health care problem. Traditionally, open procedures like the Rives-Stoppa-Sublay-Herniotomy were used as the main treatment option. With the establishment of intraperitoneal onlay-meshes (IPOM), laparoscopic management of ventral hernias was possible and the IPOM-procedure gained widespread popularity among surgeons. Due to the long-term-risks associated with the intraperitoneal placement of a synthetic mesh, the IPOM-procedure is seen more critically today by many surgeons. Alternative techniques for minimally invasive management of ventral hernia are being explored, as the retrorectus space is recognized as the optimal layer for mesh-reinforcement [1, 2]. One of the most promising approaches is the extended-totally extraperitoneal plasty (eTEP)-technique [3, 4]. During the eTEP-procedure, the retrorectus space is minimally-invasively explored and both retrorectus spaces are connected. After closure of the anterior and posterior defects, the created retromuscular space is reinforced with a synthetic mesh. The eTEP-Rives-Stoppa-Sublay-Procedure can be combined with a transversus-abdominis-release (eTAR) as posterior component separation by an experienced abdominal wall surgeon, if the hernia defect necessitates this step [3, 5].

After initial description of the eTEP-technique as a laparoscopic procedure, newer developments have gone towards robotic-assisted-eTEP-procedures. The advantage of robotic-assisted-eTEP-procedures can be mainly seen in the simplification of intracorporal suturing technique due to the increased dexterity of robotic-assisted surgery compared to conventional laparoscopic instruments. This is important, as intracorporal suturing is a main and time-consuming part of eTEP-procedures. Other benefits include 3-dimensional vision, increased precision during the preparation phase, and enhanced surgeon ergonomics [6, 7]. While there are several reports on surgical techniques on multiport-robotic-systems such as the DaVinci X or Xi-systems, only very limited reports are available for single-port robotic eTEP-procedures (SP-eTEP) on the new DaVinci SP-system (Intuitive Surgical Inc., Sunnyvale, California, United States) [8–10]. Both of the reported procedures use a suprapubic access to the preperitoneal space (suprapubic SP-eTEP). While this access might be beneficial in some cases (upper midline hernia, easy combination with bilateral “bottom-up”-TAR-procedure), we experienced in our own practice the limitations of this access due to previous surgery via a Pfannenstiel-incision, which can impede the creation of the preperitoneal space. Further limitations of this technique are that inferior midline or lateral hernia (M4-5, L3 according to EHS-classification [11]) cannot be managed via this access and problems with the maximal reach of the DaVinci SP-instruments can prohibit adequate retro-xyphoidal preparation. We therefore present our lateral-dock single-port robotic-assisted eTEP-Sublay-technique (Freiburg approach, FReTEP) in this article, which can be used synergistically to the suprapubic access for SP-eTEP-procedures.

## Methods

A feasibility study was performed in a certified medical training center (*Institut de Recherche contre les Cancers de l’Appareil Digestif* / IRCAD, Strasbourg, France) on a male human cadaver. After demonstration of technical feasibility of the approach, the technique was used on two consecutive patients with midline incisional hernia. Patients’ informed consent to undergo the surgical procedure and for publication was obtained.

## Surgical procedure

Our FReTEP-technique follows the same principles as traditional laparoscopic eTEP [12]:

1. Development of one-sided retrorectus space and port placement.
2. Cross-over of the midline (ideally) above the hernia defect anterior to the falciform ligament.
3. Connection of both retrorectus spaces.
4. Defect closure and restoration of the linea alba.
5. Mesh placement.

An example of a FReTEP-procedure is demonstrated in the supplementary video-file.

### Step 1 - Development of one-sided retrorectus space and port placement

A preoperative CT-scan or another form of cross-sectional imaging of the abdomen and hernia defects should be available for procedural planning. Surgery is performed under general anaesthesia. A urinary catheter is placed. The patient is positioned in supine position, with the left arm tucked to the side of the patient, and the operating table is flexed 20° at the level of the anterior superior iliac spine.

**Fig. 1:**
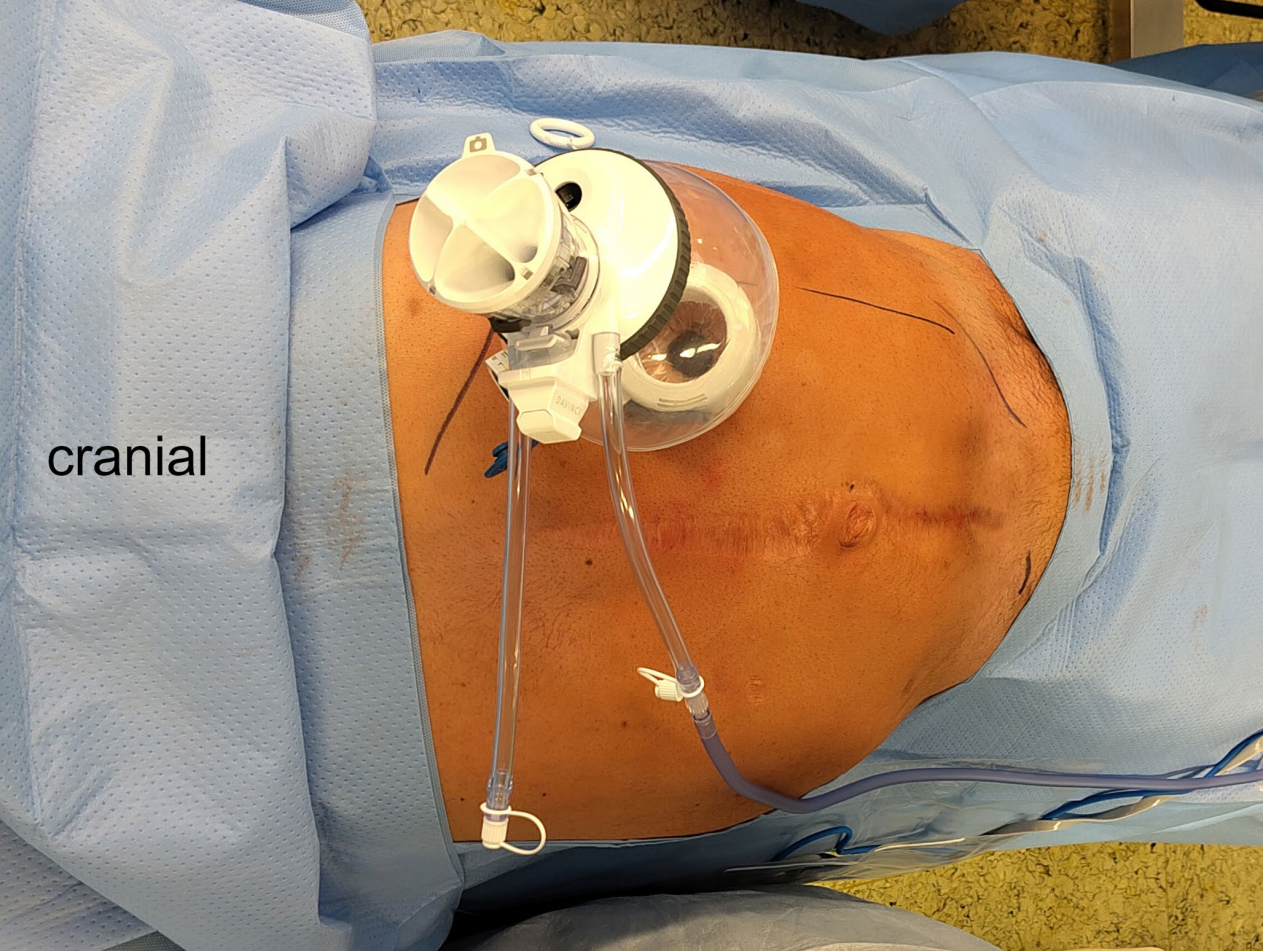
Port placement for the FReTEP-procedure. The small access port is placed in the left retrorectus space. It is important to place the incision in a location in which every part of the planned surgical field is within 27 cm of the incision. Secondly, a small distance should be left between the costal margin and the incision, so that the ring of the access port does not put pressure on the costal margin with the low operating angle of the SP-system during the procedure

**Fig. 2:**
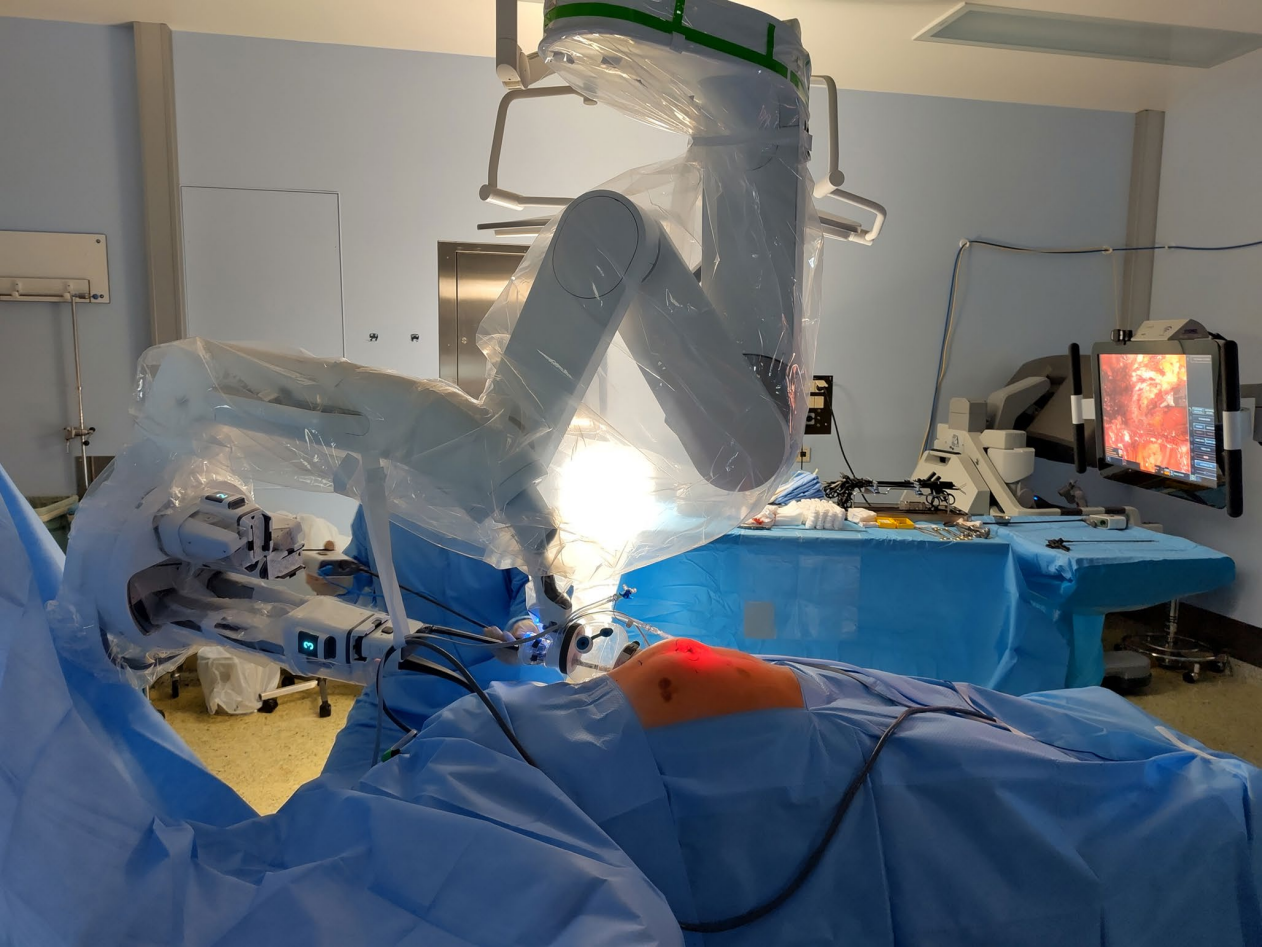
Setup of operation room and DaVinci-SP-system for FReTEP-procedure

Under ultrasonographic control, the lateral border of the retrorectus space– the linea semilunaris– and the costal margin is marked. Currently, the DaVinci SP-system has a maximal instrument reach of 27 cm with a compressed access port. This length restriction has to be accounted for in planning the location of the skin incision. Moreover, the incision has to be made as far lateral in the retrorectus space as possible to enhance SP-robotic-manoeuvrability, but a small margin of retrorectus space has to be left laterally to ensure secure placement of the access-port ring in the retromuscular space. Finally, a small distance should be left between the costal margin and the incision so that the ring of the access port does not put pressure on the costal margin with the low operating angle of the SP-system during the procedure (Figs. 1 and 2). After well-considered planning of the single-incision, a ∼ 2.7 cm long transverse incision is made and the subcutaneous tissue is transected. The anterior aspect of the anterior wall of the rectus sheath is visualised and transversally incised. Afterwards, the rectus muscle is bluntly divided and the retrorectus space is established. A small access-port (Intuitive Surgical Inc., Sunnyvale, California, United States) is placed in the retrorectus space and the retrorectus space is carefully bluntly dissected (Fig. 1). Subsequently, the DaVinci SP-system is docked and monopolar curved scissors and fenestrated bipolar forceps are used for further preparation. The camera-below position of the DaVinci SP-system is used at the beginning of the procedure.

### Step 2 - Cross-over of the midline

**Fig. 3:**
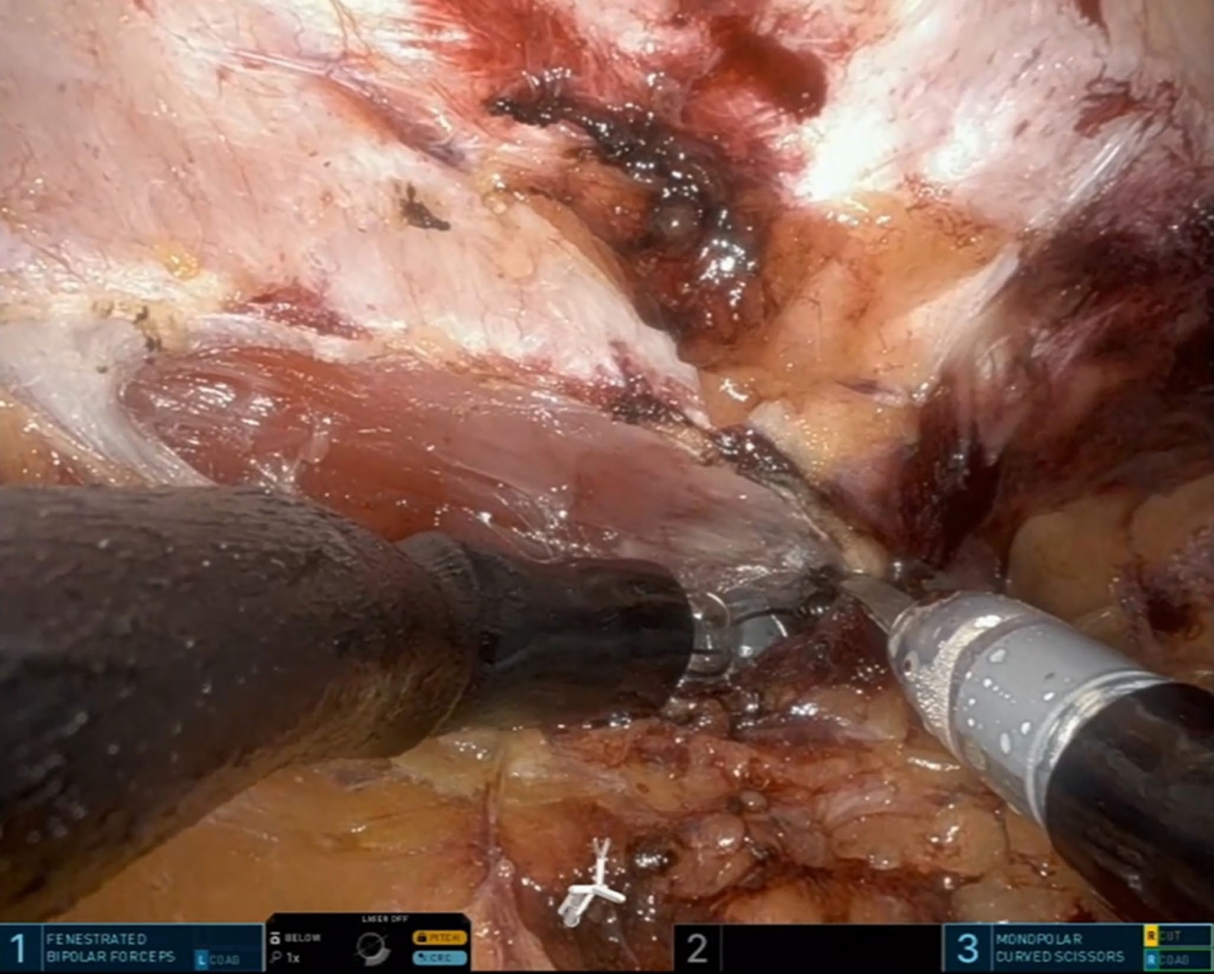
Cross-over of the midline The contralateral posterior rectus sheath is incised and the two retrorectus spaces are connected

After preparation of the (left-sided) retrorectus space, the cross-over manoeuver is performed. The medial border of the posterior rectus sheath is incised (ideally above the hernia defect) and the preperitoneal space anterior to the falciform ligament is entered. The falciform ligament is mobilized dorsally– away from the linea alba– and the contralateral rectus muscle is identified. After identification of the contralateral rectus muscle, the posterior rectus sheath of the contralateral side is incised and the two spaces are connected (Fig. 3). If necessary, this cross-over preparation can also be performed through the scar tissue/hernia defect.

### Step 3– Connection of the two retrorectus spaces– preparation of hernia sac

In the next procedural phase, the two retrorectus spaces are further connected through medial incision of the posterior rectus sheaths of both sides. The hernia is typically encountered during this phase. In primary ventral hernias the hernia content can usually be reduced easily, while in incisional hernia the hernia sac often needs to be incised and left in the subcutaneous tissue. Partial preparation of the hernia sac and preservation of the hernia sac in continuity with the posterior sheath can facilitate closure of the posterior sheath further on during the eTEP-procedure [13]. Kaudal of the hernia defect, the preparation is continued in the retromuscular plane and behind/dorsal of the linea alba with further connection of the two retrorectus spaces. The preparation is carried out as far as necessary for the treatment of the specific hernia, but preparation to the space of Retzius can be performed without any problems via the lateral-dock access.

### Step 4– Defect closure and restoration of the linea alba

After completion of the preparation of the retrorectus space, the hernia defect in the anterior wall is reconstructed using (in most cases several) 0 long-term-absorbable barbed suture (V-Loc 180, 45 cm, Medtronic, Dublin, Ireland) as a running suture. During this phase of the operation, the pressure of the pneumoperitoneum is reduced to 5–6 mmHg to relieve the tension on the sutures. After closure of the anterior wall with reconstruction of the linea alba, the posterior wall is adapted using 2 − 0 long-term-absorbable barbed suture (V-Loc 180, 30 cm, Medtronic, Dublin, Ireland). At the beginning of the closure of the posterior sheath, the camera position is switched to the camera-above position of the DaVinci SP-system. This facilitates the closure of the posterior sheath and the mesh-placement process. This setup is used until the end of the procedure.

### Step 5– Mesh placement

**Fig. 4:**
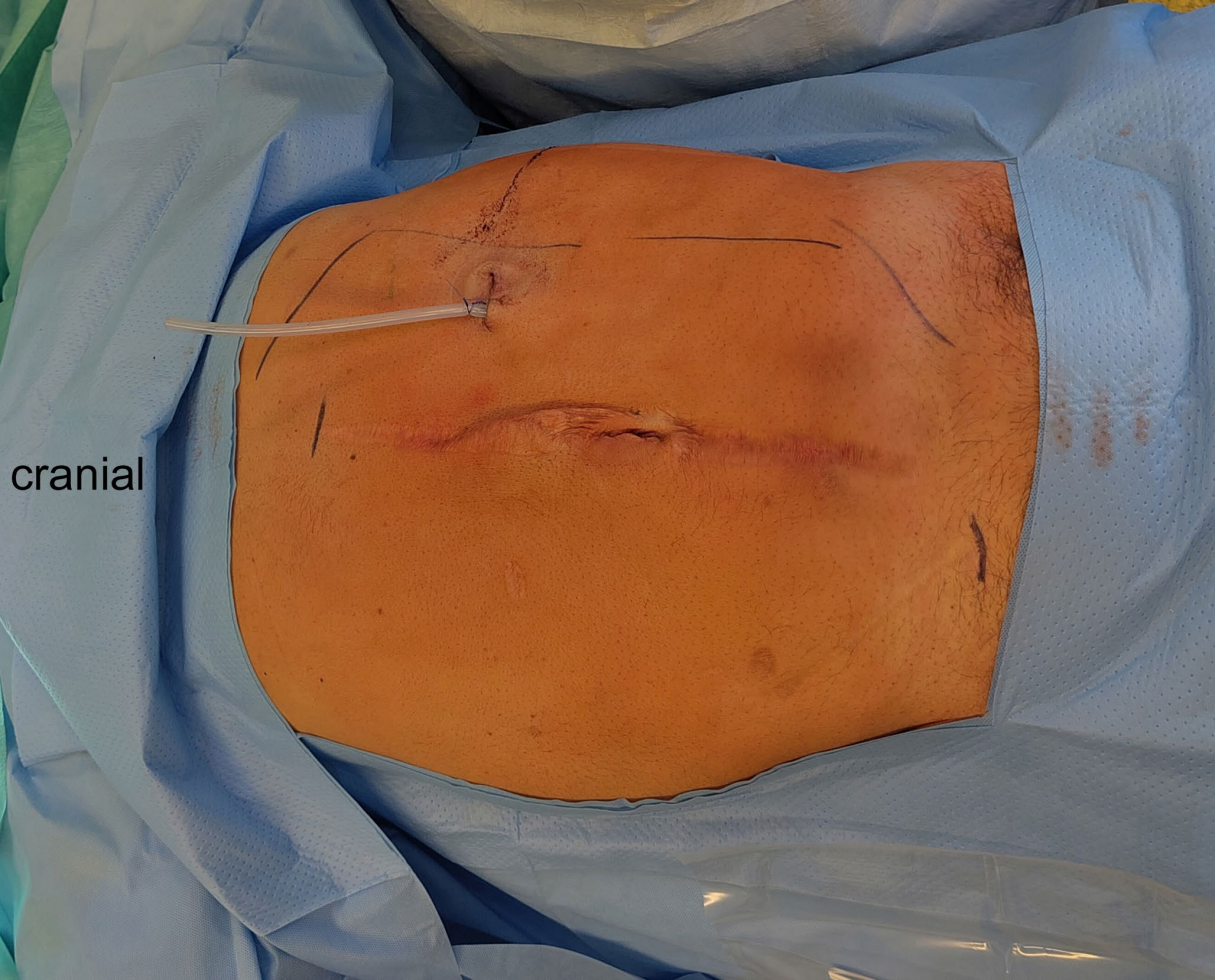
Postoperative aspect of a FReTEP-procedure

For the preparation of the last step of the procedure, a flexible ruler is placed inside the surgical space and the necessary size of the mesh is determined. A medium-weight non-absorbable polypropylene mesh (Prolene Soft, Ethicon, Raritan, New Jersey, USA) is trimmed to the right dimensions and placed flat and fixation free in the retrorectus space. A drainage is placed through the incision into the retrorectus space and the DaVinci SP-system is undocked. The drain is fixated, the anterior rectus sheath is reconstructed next to the drainage with 2 − 0 PDS running suture and the skin is closed intracutaneously. Figure 4 shows the postoperative aspect after FReTEP in the first patient with M2-4W2 incisional hernia.

## Results

In March 2025, we performed the first two cases of FReTEP in our university medical center. The first patient to undergo FReTEP was a 56-year-old male patient (BMI: 33.9 kg/m²) with M2-4W2 (Defect size (length x width): 18 × 8 cm, 144 cm³) incisional hernia after right hemicolectomy due to mesenteric ischemia via median laparotomy 12 years ago. The operation time was 286 min. We performed routine eTEP-procedure without additional eTAR-procedure and a 32 × 12 cm Prolene Soft Mesh was placed in the retrorectus space. The postoperative course was uneventful, the drain was removed on the 4th postoperative day (cumulative drain volume: 180 ml) and the patient was discharged on the same day. At the 30-day follow-up examination the patient did fine and was satisfied with the procedure, he did not show any signs of recurrence or surgical site occurrence.

The second consecutive patient to undergo FReTEP was a 63-year-old female patient (BMI: 31.2 kg/m²) with M2-4W2 (Defect size (length x width): 9 × 8 cm, 72 cm³) incisional hernia after ovarian cancer surgery via median laparotomy 3 years ago. Operation time was 192 min. We performed routine eTEP-procedure without additional eTAR-procedure and a 28 × 13 cm Prolene Soft Mesh was placed in the retrorectus space. The postoperative course was uneventful, the drain was removed on the 3rd postoperative day (cumulative drain volume: 400 ml) and the patient was discharged on the same day. At the 30-day follow-up examination the patient did fine and was also satisfied with the procedure, she did not show any signs of recurrence or surgical site occurrence.

## Discussion

In this article, we describe our adaptation of the eTEP-technique using the DaVinci SP-system to augment the robotic-surgeons armamentarium for ventral hernia repair. This is to our knowledge the first presentation of the feasibility of a lateral-dock single-port robotic-assisted eTEP-procedure.

For multiport-eTEP-procedures several docking positions have been described. The lateral dock position is the most versatile position though, enabling access to the entire midline from the xyphoid process to the pubic bone [6, 14]. In our opinion, this is true for single-port-access as well. The possibilities of this access include:

- Access to the space of Retzius and Bogros for management of inferior M4-5 hernia and lateral inferior hernia (L3).
- Single-port access to the retrorectus space in patients with impaired anatomy in the suprapubic region (e.g. prior surgery via a Pfannenstiel-incision/caesarean section).
- Access to the retroxiphoid region.
- A least contralateral TAR is easily possible, although even single-dock-bilateral TAR is feasible using the high-manoeuvrability of the DaVinci-SP-system [10]. Robotic management of parastomal hernia and lateral abdominal wall hernia L1-4 are technically possible with the same access.
- The technique offers advantages for the treatment of adipose patients, as the normal fat distribution of the abdominal wall usually involves lower thickness of the subcutaneous fat tissue below the costal margin compared to often massive pannus in the suprapubic region, which prohibit a suprapubic SP-eTEP-procedure.

Of course, disadvantages of the FReTEP compared to the suprapubic access are present:

- The main advantage of the suprapubic SP-eTEP is the near perfect ergonomics for suturing a midline hernia defect. For the suturing in the suprapubic-SP-eTEP-technique, no extreme angulation in the “elbows” of the SP-robot-instruments is necessary and the suturing-process is done with ease in this setting. Contrary to this, suturing has to be done in the FReTEP with an angulation in the “elbows”. Stiches below the access-port can be done with the right hand (given that an access into the left rectus sheath was chosen), while stiches above the access-port will usually have to be made using the left-hand instrument due to movement restrictions of the SP-instruments. For transverse incisions, of course, these advantages/disadvantages interchange.
- Bilateral TAR can be performed easily via the suprapubic access.
- A further technical limitation is– at least in the beginning of the application of the technique - that a certain width ≥ 6.5–7 cm of the retrorectus space is needed for placement of the access-port. A smaller diameter rectus sheath can be entered, if the hernia defect is considerably distant from the access-site and no suturing will be needed close to the access-port. Otherwise, the internal ring of the access-port will hinder well placed stiches close to the access-port.

In our opinion, both accesses should be regularly performed by any surgeon performing SP-eTEP-procedures, and the access of choice should be tailored individually to the specific patient and specific hernia. Therefore, the lateral- and suprapubic access can be used in a synergistic manner by DaVinci SP-surgeons to take advantage from the best port placement for a specific hernia location and other patient’s characteristics.

Whether SP-eTEP has any advantages over Multiport-eTEP will be another matter of debate. Possible advantages could be improved cosmetics and possibly less postoperative pain due to reduced size of the entirety of the incisions. A side-effect to be aware of in the future is the incidence of trocar site hernia after SP-(eTEP-)procedures. Due to the slightly larger size of the single incision, there is an increased risk for trocar site hernia-development, as has been shown previously for single-site-surgery in conventional laparoscopy as well [15]. In our opinion, this risk appears to be theoretically low in SP-eTEP-procedures, especially for the FReTEP, because (1) the posterior rectus sheath stays intact and (2) the site of the single-port-access is reinforced with the synthetic mesh as well. Nevertheless, the incidence will have to be monitored and reported in future medium-and long-term-studies reporting SP-eTEP-outcomes.

The limitation of our study is of course the lack of treatment data of a larger cohort demonstrating the safety of the procedure. This is the first report of the novel access for SP-eTEP. We have no reason to assume that there are risks specific to our access to the eTEP-procedure, but we will process the cohort of upcoming lateral dock- and suprapubic SP-eTEP-patients scientifically to generate the necessary data.

In conclusion, we present a novel access for SP-eTEP-procedures. Via the FReTEP-procedure a wide variety of hernia defect- and patient characteristics can be treated. Future evaluation of the technique is necessary to provide further safety and long-term efficacy results.

## Electronic supplementary material

Below is the link to the electronic supplementary material.

Supplementary Material 1

Supplementary Material 2

Supplementary Material 3

Supplementary Material 4

Supplementary Material 5

Supplementary Material 6

## Data Availability

Further details regarding the presented technique are available from the corresponding author upon reasonable request.
